# Resources Within: Between Resident Ties and Links to Health Among Older Subsidized Housing Residents

**DOI:** 10.1093/geroni/igab046.521

**Published:** 2021-12-17

**Authors:** Noah Webster

**Affiliations:** University of Michigan, Ann Arbor, Michigan, United States

## Abstract

Increasing evidence points to the importance of non-family ties in promoting health among older adults. Less is known though about these ties within the context of subsidized housing. In this study we examine prevalence of social ties between residents and examine links to health. Data were collected through interviews conducted with 39 residents age 62 and older living in a subsidized housing community in Southeast Michigan. Residents reported knowing on average 10 (SD=6.5) other residents, and nominated three (SD=4.2) residents into their close social networks. Residents who reported getting out of the community less often and those with one or more health limitations nominated significantly more residents into their network. Also, getting out of one’s apartment more often was associated with knowing more residents in the community. Findings highlight between resident ties may serve as an important resource for those geographically restricted and may be useful to integrate into interventions.

